# Supplementary material for: Multivalent interactions between molecular components involved in fast endophilin mediated endocytosis drive protein phase separation
Source: Nat Commun. 2022 Aug 26;13:5017. doi: 10.1038/s41467-022-32529-0 (PMC9418313; doi:10.1038/s41467-022-32529-0)
Supplement: Supplementary file 3 — Description of Additional Supplementary Files [file 41467_2022_32529_MOESM3_ESM.docx]

**Descriptions of Additional Supplementary Files**

**Supplementary Movie 1:** Demonstration of tubule contraction on a GUV. GUV shows dynamic, micron long tubules in the presence of endophilin (green) before binding of LPD^850-1250^. Introduction of LPD^850-1250^ (magenta) causes contraction of tubules indicating membrane adherence.

**Supplementary Movie 2:** Demonstration of an increase in the membrane tension upon introduction of LPD (magenta) to endophilin (green) coated, tubulated GUVs. Enhanced membrane tension upon addition of LPD pulls two GUVs tethered via tubules closer to each other.
