# Supplementary material for: Multivalent interactions between molecular components involved in fast endophilin mediated endocytosis drive protein phase separation
Source: Nat Commun. 2022 Aug 26;13:5017. doi: 10.1038/s41467-022-32529-0 (PMC9418313; doi:10.1038/s41467-022-32529-0)
Supplement: Supplementary file 6 — Reporting Summary [file 41467_2022_32529_MOESM6_ESM.pdf]

Corresponding author(s): Tobias Baumgart

Last updated by author(s): Jul 31, 2022

Reporting Summary

Nature Portfolio wishes to improve the reproducibility of the work that we publish. This form provides structure for consistency and transparency in reporting. For further information on Nature Portfolio policies, see our Editorial Policies and the Editorial Policy Checklist.

Statistics

For all statistical analyses, confirm that the following items are present in the figure legend, table legend, main text, or Methods section.

n/a	Confirmed	
☐	☒	The exact sample size (*n*) for each experimental group/condition, given as a discrete number and unit of measurement
☐	☒	A statement on whether measurements were taken from distinct samples or whether the same sample was measured repeatedly
☐	☒	The statistical test(s) used AND whether they are one- or two-sided<br>*Only common tests should be described solely by name; describe more complex techniques in the Methods section.*
☐	☒	A description of all covariates tested
☒	☐	A description of any assumptions or corrections, such as tests of normality and adjustment for multiple comparisons
☐	☒	A full description of the statistical parameters including central tendency (e.g. means) or other basic estimates (e.g. regression coefficient) AND variation (e.g. standard deviation) or associated estimates of uncertainty (e.g. confidence intervals)
☐	☒	For null hypothesis testing, the test statistic (e.g. *F*, *t*, *r*) with confidence intervals, effect sizes, degrees of freedom and *P* value noted<br>*Give P values as exact values whenever suitable.*
☒	☐	For Bayesian analysis, information on the choice of priors and Markov chain Monte Carlo settings
☒	☐	For hierarchical and complex designs, identification of the appropriate level for tests and full reporting of outcomes
☐	☒	Estimates of effect sizes (e.g. Cohen's *d*, Pearson's *r*), indicating how they were calculated

Our web collection on statistics for biologists contains articles on many of the points above.

Software and code

Policy information about availability of computer code

Data collection	Confocal images were recorded using Olympus FV31S-SW software (version 2.1.1.98).
Data analysis	ImageJ (version 1.52a) was primarily used for the analysis of confocal imaging data. The FRAP data analysis was performed using Microsoft Excel (version 2013 and 2019) and Origin Pro (version 2021). Statistical analysis (Student's t-test, two-tailed) was performed in Microsoft Excel. Autocorrelation analysis was performed using normcorr2 plugin (https://www.mathworks.com/help/images/ref/normxcorr2.html) in MATLAB (versions 2019b and 2020a). Home-built analysis codes used to generate radial autocorrelation, cross-correlation plots, and the GUV image analysis are written in Python (version 3.7.1) using OpenCV package (version 4.4.0.44).<br><br>Code availability: The MATLAB and Python based codes for correlation analysis and GUV analysis are uploaded to Github repository (https://github.com/Baumgartlab/Corrlation-analysis-and-GUV-analysis).

For manuscripts utilizing custom algorithms or software that are central to the research but not yet described in published literature, software must be made available to editors and reviewers. We strongly encourage code deposition in a community repository (e.g. GitHub). See the Nature Portfolio guidelines for submitting code & software for further information.

Data

Policy information about availability of data

All manuscripts must include a data availability statement. This statement should provide the following information, where applicable:

- Accession codes, unique identifiers, or web links for publicly available datasets
- A description of any restrictions on data availability
- For clinical datasets or third party data, please ensure that the statement adheres to our policy

All processed data generated and analyzed for this study are included in the article and the supporting information. Raw image files have been uploaded to Figshare repository (https://doi.org/10.6084/m9.figshare.20388570.v1). Source data are provided with this paper.

Field-specific reporting

Please select the one below that is the best fit for your research. If you are not sure, read the appropriate sections before making your selection.

[x] Life sciences [ ] Behavioural & social sciences [ ] Ecological, evolutionary & environmental sciences

For a reference copy of the document with all sections, see nature.com/documents/nr-reporting-summary-flat.pdf

Life sciences study design

All studies must disclose on these points even when the disclosure is negative.

Sample size	The sample size were chosen such that significant statistical information (mean, standard deviation, standard error of mean, and P values) could be derived. The exact sample size (e.g. the number of droplets) are mentioned in the figure legends.
Data exclusions	No data were excluded from the analysis.
Replication	The phase boundary determination experiments were repeated at least in duplicate. All other experiments were replicated at least in triplicate to ensure reproducibility and to estimate mean, standard deviation, and standard error of mean values. All replicates produced similar observation within the limit of experimental error limits.
Randomization	For our droplet FRAP studies, the droplets were randomly selected without any biases. For all other studies, data analysis was performed on the entire dataset collected.
Blinding	Blinding was not relevant in our completely in vitro study. All judgments made were based on statistical significance tests.

Reporting for specific materials, systems and methods

We require information from authors about some types of materials, experimental systems and methods used in many studies. Here, indicate whether each material, system or method listed is relevant to your study. If you are not sure if a list item applies to your research, read the appropriate section before selecting a response.

Materials & experimental systems

n/a	Involved in the study	
[x]	[ ]	Antibodies
[x]	[ ]	Eukaryotic cell lines
[x]	[ ]	Palaeontology and archaeology
[x]	[ ]	Animals and other organisms
[x]	[ ]	Human research participants
[x]	[ ]	Clinical data
[x]	[ ]	Dual use research of concern

Methods

n/a	Involved in the study	
[x]	[ ]	ChIP-seq
[x]	[ ]	Flow cytometry
[x]	[ ]	MRI-based neuroimaging
